# Post-COVID-19 neuropsychiatric manifestations among COVID-19 survivors suffering from migraine: a case–control study

**DOI:** 10.1186/s10194-022-01468-y

**Published:** 2022-08-12

**Authors:** Rehab Magdy, Alaa Elmazny, Shaimaa H. Soliman, Eman H. Elsebaie, Sara H. Ali, Ali M. Abdel Fattah, Mahmoud Hassan, Ahmed Yassien, Noha A. Mahfouz, Radwa M. Elsayed, Wael Fathy, Hoda M. Abdel-Hamid, Jehan Mohamed, Mona Hussein

**Affiliations:** 1 Neurology Department, Faculty of Medicine, Cairo University, Cairo, Egypt; 2 Internal Medicine Department, Arabian Gulf University, Manama, Bahrain; 3 Department of Public Health and Community Medicine, Faculty of Medicine, Cairo University, Cairo, Egypt; 4 Department of Otolaryngology, Faculty of Medicine, Beni-Suef University, Beni- Suef, Egypt; 5 Department of Gastroenterology, Hepatology, and Endemic Medicine, Faculty of Medicine, Beni-Suef University, Beni- Suef, Egypt; 6 Department of Internal Medicine, Faculty of Medicine, Beni-Suef University, Beni- Suef, Egypt; 7 Department of Critical Care Medicine, Beni-Suef University, Beni-Suef, Egypt; 8 Department of Psychiatry Faculty of Medicine, Cairo University, Cairo, Egypt; 9 Department of Family Medicine, Faculty of Medicine, Cairo University, Cairo, Egypt; 10 Department of Anaesthesia, Surgical ICU and Pain Management, Beni-Suef University, Beni-Suef, Egypt; 11 Department of Chest Diseases, Faculty of Medicine, Cairo University, Cairo, Egypt; 12 Neurology department, Beni-Suef University, Beni-Suef, Egypt

**Keywords:** SARS-CoV-2, Migraine, Long COVID-19, Neuropsychiatric symptoms, Headache

## Abstract

**Background:**

The burden of post-coronavirus disease (COVID)-19 symptoms has been increasing and is of great concern in patients with pre-existing chronic medical conditions.This study aimed to delineate the post-COVID-19 neuropsychiatric symptoms among migraine patients compared to the non-migraine control group.

**Methods:**

Two groups, each of 204 COVID-19 survivors, were enrolled in the study after 3 months of severe acute respiratory syndrome coronavirus-2 (SARS-CoV-2) infection, one group fulfilling the episodic migraine criteria and the other serving as a matching control group. Subjects were evaluated through an in-person interview for post-COVID-19 neuropsychiatric symptoms, including detailed headache patterns and severity, using the visual analogue scale.

**Results:**

The Frequency of headache during the acute phase of COVID-19 was more frequent in migraine patients (OR = 1.60, 95%CI = 1.04–2.45, *P-*value = 0.031). The reported significant post-COVID-19 neuropsychiatric symptoms in migraine patients compared to controls were fatigue (OR = 1.662, 95%CI = 1.064–2.596, *P-*value = 0.025), anosmia/hyposmia (OR = 2.06, 95%CI = 1.164- 3.645, *P-*value = 0.012), cacosmia (OR = 2.663, 95%CI = 1.145–6.195, *P-*value = 0.019), depression (OR = 2.259, 95%CI = 1.284- 3.975, *P-*value = 0.004), anxiety (OR = 3.267, 95%CI = 1.747- 6.108, *P-*value ≤ 0.001), insomnia (OR = 2.203, 95%CI = 1.298- 3.739, *P-*value = 0.003), and headache (OR = 3.148, 95%CI = 1.616–6.136, *P-*value =  ≤ 0.001).While there was no statistically significant difference between migraine patients and controls regarding the post-COVID-19 functional status score (*P-*value = 0.102). The pattern of post-COVID-19 headache was reported as chronic headache transformation in 17.6% of the migraine group, with the median intensity rate being 5.5 and IQR (3–7). In the control group, 14% experienced chronic headache attributed to systemic viral infection with a median intensity rate of 2 and IQR (2–5), while 12% experienced a new daily persistent headache with a median intensity of 5 and IQR (1–6).

**Conclusion:**

The study highlighted the importance of follow-up migraine patients upon recovery from COVID-19 infection, being more vulnerable to post-COVID-19 symptoms.

## Introduction

Since COVID-19 has been declared a pandemic, healthcare systems worldwide face increasing challenges, including the long-term consequences of this infection [1]. A large meta-analysis by Fernández-de-Las-Peñas et al. [2] revealed that more than 60% of COVID-19 survivors experienced post-COVID-19 symptoms.

The exact pathophysiological mechanisms behind the post-COVID-19 manifestations are not settled. The direct effects of viral infection, the inflammatory response and the immune reaction, besides the psychological factor, might be all involved in the aetiological process. The magnitude of neuroinflammatory response would be more robust in predisposed subjects as in the case of patients with chronic conditions [3, 4].

Mounting evidence exists that people with pre-existing chronic medical conditions would be more likely to have severe COVID-19 infection and, therefore, more likely to develop post-COVID-19 symptoms [5, 6]. Whether people with migraines would be more likely to have post-COVID-19 condition has not been studied before.

Migraine was described by Karsan, Goadsby [7] as a neural disorder of brain function, being associated in a bidirectional manner with other disorders, such as chronic fatigue, mood, cognitive and sleep disorders, and other pain conditions. Specifically, migraine patients are more prone to psychiatric disorders resulting in enhanced psychosocial impairment raising the importance of psychological evaluation of patients with migraine [8]. All these neuropsychiatric symptoms are encountered in the definition of the post-COVID-19 condition [3].

A case–control study conducted by Fernández-de-Las-Peñas, Gómez-Mayordomo [9] revealed that those who had a headache in the acute phase of infection were more prone to develop long-term post-COVID-19 symptoms.

Migraine sufferers were more likely to develop headaches during the acute phase of COVID-19 infection [10]. However, surprisingly, they were not reported to have headaches in the post-COVID phase. They only exhibited long-term fatigue as post-COVID-19 sequelae [11].

Based on these findings, we hypothesized that COVID-19 survivors with a history of migraine might develop some post-COVID-19 neuropsychiatric symptoms more frequently than those without a history of migraine.

Understanding the burden of post-COVID-19 neuropsychiatric manifestations among migraine patients will improve rehabilitation services, which meet the emerging needs of patients with migraine who had been infected with SARS-CoV-2. Hence, this study aims to characterize the post-COVID-19 neuropsychiatric manifestations and their burden on migraine patients compared to the non-migraine control group.

## Methods

### Study design and participants

This case–control study was conducted at Beni-Suef University Hospital, Egypt, one of the largest tertiary academic hospitals serving both rural and urban areas in upper Egypt. The study population included all adult patients with a confirmed COVID-19 diagnosis seen in the COVID-19 outpatient clinic or discharged alive after hospitalization due to COVID-19 infection between June 1, 2021, and March 30, 2022, during the third wave of the pandemic. We adhered to the strengthening in the Reporting of Observational Studies in Epidemiology (STROBE) guidelines for case–control studies [12].

### Recruitment and initial assessment phase

Patients were contacted by telephone after obtaining their demographics and medical history from their health records 3-months after the acute phase of COVID-19. They were interviewed regarding the symptoms presented during the acute phase and symptoms that persisted, returned or were ongoing after it, according to the criteria for post-COVID-19 conditions (as defined by the WHO [13]). Patients were asked about fatigue, pain, headache, anosmia/hyposmia, cacosmia, ageusia/hypogeusia, vertigo, tinnitus, cognitive dysfunction, depression, anxiety, or insomnia, and then invited for a face-to-face interview for objective evaluation.

History of pre-COVID headaches was obtained from the patients’ health records. In addition, all patients were systematically asked about their headache history, symptomatology, and the number of headache days per month before contracting COVID-19 during the telephone interview.

According to the WHO classification, clinical /radiological signs of pneumonia and the need for oxygen were the factors that grade the infection severity into mild, moderate, or severe [14].

Patients were also categorized according to their vaccination status prior to COVID-19 infection into fully vaccinated and non-vaccinated, conforming to the Centers for Disease Control and Prevention (CDC) definitions [15].

Patients were eligible for enrollment if they: 1) were older than 18 years, 2) had a confirmed history of COVID-19 diagnosis by reverse transcription-polymerase chain reaction (RT-PCR) by nasal and oropharynx swabs. Exclusionary criteria included: 1) refusal to participate in the study, 2) receiving single-dose vaccination prior to COVID-19 infection [with the exception given to Johnson and Johnson vaccine], 3) the assessed neuropsychiatric symptoms could be explained by a pre-existing medical condition, 4) Inability to reach the patient after at least three attempts using all available contact options.

**Fig. 1:**
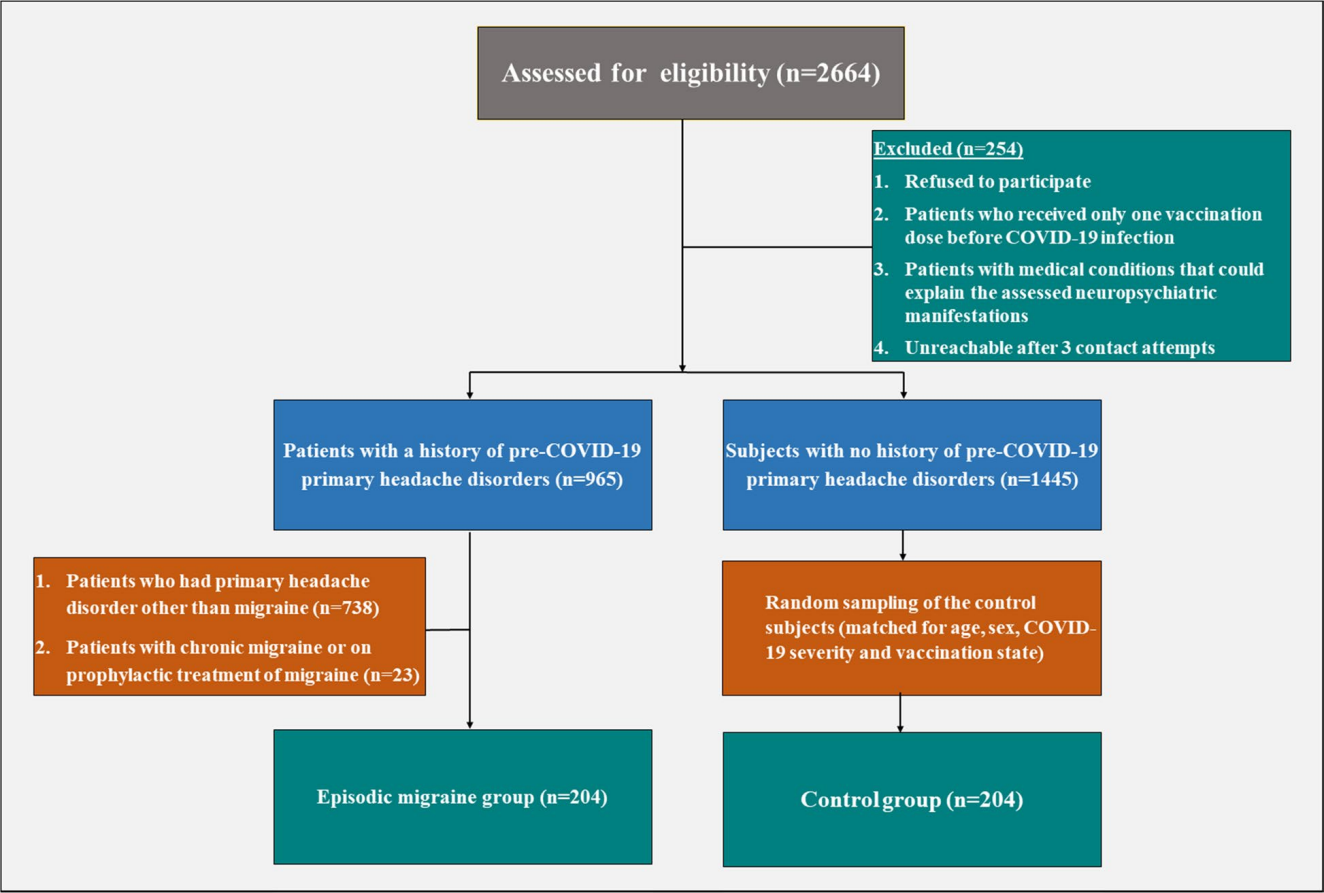
Flow diagram for the included and the excluded patients and controls

Through convenience sampling, a group of COVID-19 survivors with a confirmed history of pre- COVID episodic migraine according to the third edition of the international classification of headache disorders ICHD-3 criteria [16] were recruited as migraine headache sufferers. Another group of COVID-19 survivors with no history of any primary headache disorders preceding COVID-19 infection served as controls via randomized sampling after matching for age, gender, COVID-19 severity and vaccination status. The patient’s flow diagram is outlined in Fig. 1.

### Face-to-face assessment of post-COVID-19 symptoms

A detailed general, neurological and otolaryngological examination was done for all patients who attended the face-to-face interview. Post-viral fatigue was confirmed based on the Institute of Medicine diagnostic criteria [17]. A cut off value of 4/10 in the Douleur Neuropathique en 4 Questions (DN4) [18], was used to delineate patients reporting neuropathic pain. The diagnostic and Statistical Manual of Mental Disorders (DSM-5) [19] was applied for diagnosing insomnia, depressive and anxiety disorders. The Montreal Cognitive Assessment (MoCA), with a cut-off score of 26 [20], was used to define the diagnosis of post-COVID-19 cognitive dysfunction (PCCD). And finally, the post-COVID-19 Functional Status scale [21] was used to assess the functional limitations caused by all symptoms.

**Fig. 2:**
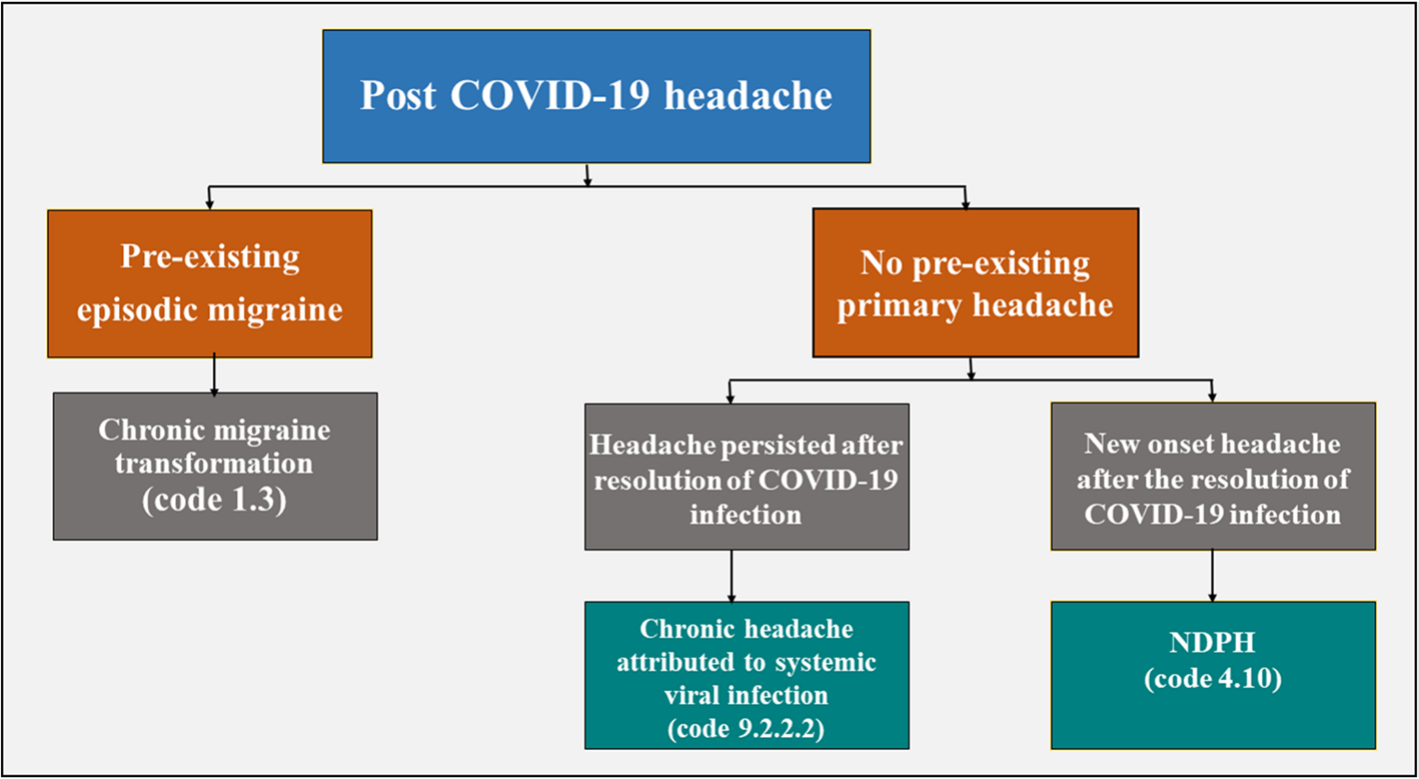
Patterns of post-COVID-19 headache

Diagnosis of post-COVID-19 headache was established if any of the following three clinical patterns were identified [22]. The first pattern corresponds to the diagnostic code of chronic migraine (code 1.3) in patients who had episodic migraine before contracting COVID-19. For the control group, either of the following patterns was described. If the headache persisted beyond the acute viral stage despite the resolution of other COVID-19 symptoms, it would correspond to the diagnostic code of chronic headache attributed to systemic viral infection (code 9.2.2.2). On the other hand, if the headache had a clear onset after the resolution of acute viral infection, in the absence of a history of headache during COVID-19 infection and followed a non-remitting course, it would correspond to the diagnostic code of New daily persistent headache (NDPH) (code 4.10) (Fig. 2). Patients with post-COVID-19 headaches were requested to rate the intensity of their headaches using the visual analogue scale (VAS).

### Ethical statement

Oral consent was obtained from all participants over the phone after the study purpose was explained, and written informed consents were signed by patients who attended the face-to-face interview. The study was performed in accordance with the Helsinki declaration, and the Beni-Suef University hospital research ethical committee approved the study (approval number is FMBSUREC/07062022/Tawfik).

### Sampling

The sample size was calculated using Epi calc 2000, version 1.02, 1997. Based on 10% prevalence rate of migraine [23], a null hypothesis of 17%, and an alpha level of significance of 0.05, a total sample size of at least 199 participants was required to achieve a statistical power of 80%.

### Statistical analysis

The data were analyzed using SPSS version 25. We used Kolmogorov–Smirnov test to check the normality of the quantitative variables (age & VAS). Both variables were not normally distributed. So, they were expressed as the median and interquartile range (IQR). Categorical variables were expressed as numbers and percentages. Mann–Whitney test was used to compare migraine patients and the control group in age. Chi-square test was used to compare migraine patients and the control group in terms of sex, COVID-19 symptoms and severity, vaccination state before COVID-19 infection, and post-COVID-19 neuropsychiatric manifestations. The *P-*values were adjusted for multiple testing by performing the Benjamini–Hochberg procedure. A *P-*value less than 0.05 was considered statistically significant. All tests were two-tailed.

## Results

### Demographics and COVID-19 symptoms in migraine patients and controls

This case–control study was conducted on 204 COVID-19 survivors fulfilling diagnostic criteria for episodic migraine (before being infected with COVID-19) (migraine group) and 204 age and sex-matched COVID-19 survivors not known to have any primary headache disorder (control group) (*P-*value = 0.054, 0.158 respectively). In the migraine group, 26 (12.75%) patients had an aura, while178 (87.25%) didn’t have an aura.

Regarding the symptoms reported during the COVID-19 illness, the occurrence of COVID-19-related headache was significantly higher in migraine patients than in the control group (*P-*value = 0.031). Whereas there was no statistically significant difference between migraine patients and controls regarding the occurrence of the other COVID-19 symptoms, including fever, cough/dyspnea, vomiting/diarrhea, fatigue, ageusia/hypogeusia, or anosmia/hyposmia (*P-*value = 0.171, 0.053, 0.206, 0.336, 0.62, 0.273 respectively).

Also, there was no statistically significant difference between migraine patients and controls regarding COVID-19 severity or state of vaccination before COVID-19 infection (*P-*value = 0.124, 0.701, respectively) (Table 1).

**Table 1:** Demographics and COVID-19 symptoms in migraine patients and controls

		Migraine Patients (*n* = 204)	Controls (*n* = 204)	*P-*value	Odds ratio	95% CI	
						Lower	Upper
Age [(median (IQR)]		30 (15.5- 43)	36 (30- 42.75)	0.054			
Sex [n (%)]	Male	41 (20.1%)	53 (26.0%)	0.158	1.395	0.877	2.219
	Female	163 (79.9%)	151 (74.0%)				
COVID-19 symptoms [n (%)]	Fever	130 (63.7%)	143 (70.1%)	0.171	0.749	0.495	1.134
	Cough/dyspnea	136 (66.7%)	117 (57.4%)	0.053	1.487	0.995	2.223
	Vomiting/diarrhea	73 (35.8%)	61 (29.9%)	0.206	1.306	0.863	1.977
	Fatigue	164 (80.4%)	156 (76.5%)	0.336	1.262	0.786	2.025
	Headache	153 (75%)	133 (65.2%)	0.031*	1.602	1.044	2.458
	Ageusia/hypogeusia	97 (47.5%)	92 (45.1%)	0.62	1.104	0.748	1.629
	Anosmia/hyposmia	119 (58.3%)	108 (52.9%)	0.273	1.244	0.842	1.84
	others	19 (9.3%)	18 (8.8%)	0.863	1.061	0.54	2.086
COVID-19 severity ^a^ [n (%)]	Mild	177 (86.8%)	165 (80.9%)	0.124			
	Moderate	23 (11.3%)	28 (13.7%)				
	Severe	4 (2%)	11 (5.4%)				
Full vaccination before COVID-19 infection		36 (17.6%)	39 (19.1%)	0.701	0.907	0.549	1.497

*P-*value ≤ 0.05 is considered significant

^a^Risk Estimate statistics cannot be computed. They are only computed for a 2*2 table

### Post COVID-19 neuropsychiatric manifestations in migraine patients and controls

The occurrence of post-COVID-19 fatigue, anosmia/hyposmia, cacosmia, depression, anxiety, insomnia, and headache were significantly higher among migraine patients than in the control group (*P-*value = 0.025, 0.012, 0.019, 0.004, ≤ 0.001, 0.003, and ≤ 0.001 respectively). In contrast, there was no statistically significant difference between migraine patients and controls regarding the occurrence of post-COVID-19 neuropathic pain, ageusia/hypogeusia, dizziness, tinnitus, or cognitive dysfunction (*P-*value = 0.089, 0.303, 0.205, 0.377, 0.869 respectively) (Table 2).

**Table 2:** Post-COVID-19 neuropsychiatric manifestations in migraine patients and controls

		Migraine Patients (*n* = 204)	Controls (*n* = 204)	*P-*value	Odds ratio	95% CI	
						Lower	Upper
Post COVID-19 neuropsychiatric manifestations [n (%)]	Fatigue	64 (31.4%)	44 (21.6%)	0.025*	1.662	1.064	2.596
	Neuropathic pain	35 (17.2%)	23 (11.3%)	0.089	1.63	0.925	2.871
	Anosmia/hyposmia	39 (19.1%)	21 (10.3%)	0.012*	2.06	1.164	3.645
	Cacosmia	20 (9.8%)	8 (3.9%)	0.019*	2.663	1.145	6.195
	Ageusia/hypogeusia	30 (14.7%)	23 (11.3%)	0.303	1.357	0.758	2.427
	Dizziness	34 (16.7%)	25 (12.3%)	0.205	1.432	0.82	2.5
	Tinnitus	15 (7.4%)	20 (9.8%)	0.377	0.73	0.363	1.47
	Cognitive dysfunction	21 (10.3%)	20 (9.8%)	0.869	1.056	0.554	2.014
	Depression	42 (20.6%)	21 (10.3%)	0.004*	2.259	1.284	3.975
	Anxiety	42 (20.6%)	15 (7.4%)	≤ 0.001*	3.267	1.747	6.108
	Insomnia	48 (23.5%)	25 (12.3%)	0.003*	2.203	1.298	3.739
	Headache	36 (17.6%)	13 (6.4%)	≤ 0.001*	3.148	1.616	6.136

*P-*value ≤ 0.05 is considered significant

The P values were adjusted for multiple testing using Benjamini and Hochberg procedure

**Fig. 3:**
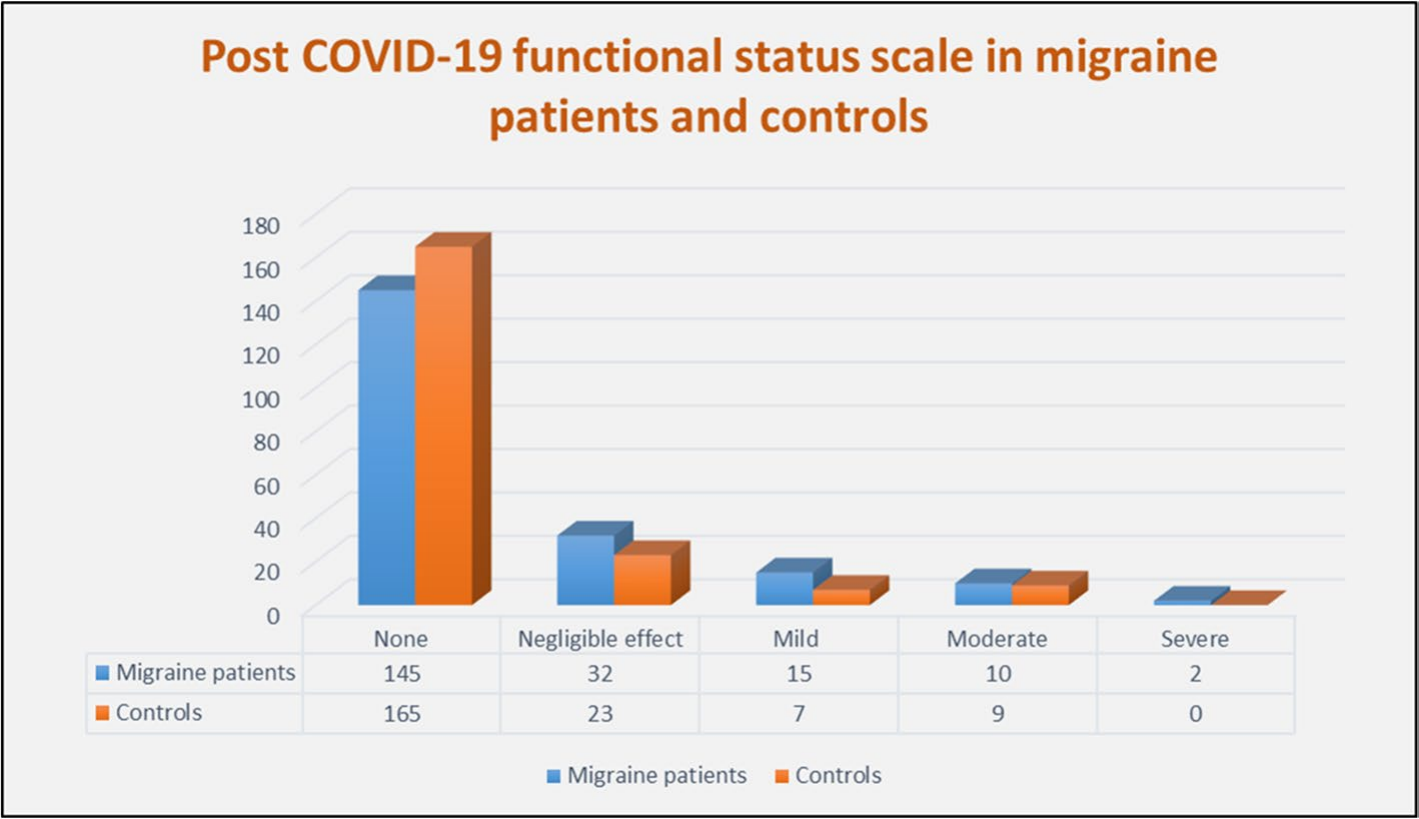
Post-COVID-19 functional status scale in migraine patients and controls

Regarding the post-COVID-19 functional status, there was no statistically significant difference between migraine patients and controls in the scores of FSS (*P-*value = 0.102) (Fig. 3).

### Characteristics and patterns of post-COVID-19 headache

In the migraine group, 36 (17.6%) patients experienced chronic headache transformation, described as a migraine-like character by 30 (83.3%) patients, whereas only 6 (16.7%) patients reported tension-like character. Patients in the migraine group rated their headache intensity with a median of 5.5 and IQR (3–7) (Table 3).

**Table 3:** Character and intensity of the different patterns of post-COVID-19 headache

		Migraine Patients	Controls	
		Chronic migraine transformation (*n* = 36)	Headache attributed to systemic viral infection (*n* = 7)	New daily persistent headache (*n* = 6)
Headache character [n (%)]	Migraine-like character	30 (83.3%)	4 (57.1%)	3 (50%)
	Tension -like character	6 (16.7%)	3 (42.9%)	3 (50%)
Headache intensity by VAS [(median (IQR)]		5.5 (3–7)	2 (2–5)	5 (1–6)

*VAS* Visual analogue scale

In the control group, seven patients (3.43%) experienced chronic headache attributed to systemic viral infection{4 patients had a migraine-like character, and 3 had a tension-like character}, with a median intensity of 2 and IQR (2–5). Also, six patients (12%) experienced a new daily persistent headache{3 patients had a migraine-like character, and 3 had a tension-like character}, with a median intensity of 5 and IQR (1–6) (Table 3).

## Discussion

It is essential to study and quantify the burden of the post-COVID-19 symptoms in migraine patients to shape specific recommendations for such population upon recovery from infection. To our knowledge, this is the first study that investigated a broad range of post-COVID-19 symptoms in patients with a confirmed diagnosis of migraine. Among these symptoms, it was found that fatigue, anxiety, depression, insomnia, headache, and olfactory impairment were affected more in migraine patients than those without migraine. However, no difference in limitations of daily living activities was found between the two groups.

First, in order to prove or refute our hypothesis that migraine patients are more likely to have post-COVID-19 problems than others, several factors known to increase the risk of post-COVID-19 symptoms have been considered. In this study, the migraine and control groups were matched for age, gender, COVID-19 symptoms, infection severity, and vaccination status prior to infection, all of which have been shown to increase the risk of post-COVID-19 symptoms in previous reports [24, 25]. However, the matching could not be achieved regards headache during acute infection. In agreement with previous studies, migraine patients exhibited headache during the acute phase more frequently than those without headaches.

In the context of the current findings, as some post‐COVID-19 symptoms were more prevalent among migraine patients than those without migraine, we propose that shared neurobiological mechanisms are likely involved in the link between migraine and post-COVID-19.

Post-COVID-19 olfactory alterations are primarily attributed to neuroinflammation of the olfactory bulb being more robust in predisposed subjects [26]. Migraine patients already have a higher baseline rate of olfactory bulb atrophy than healthy individuals in the same age range [27]; this may make migraine patients more susceptible to olfactory impairment as COVID-19 sequelae.

It is broadly accepted that a prolonged state of low-grade neuroinflammation mediated by elevated IL-1Beta and IL-6, along with central mechanisms represented by hypothalamic involvement and altered serotoninergic level, would eventually lead to post-COVID-19 fatigue [28–30]. Intriguingly, all these factors were previously described in migraine patients relative to healthy controls [7, 31], rendering them susceptible to chronic fatigue syndrome [32].

Of particular recent interest in pathogenic mechanisms of neuropsychiatric sequelae of COVID-19 infection is the role of biogenic amines. It has been demonstrated that cytokine storm induced by COVID-19 infection potentially may contribute to suppressed serotonin levels [33], a well-established biological basis of depression and anxiety [34]. Given that low cerebral serotonin level during headache-free periods is a common feature in migraine patients [35], a high frequency of post-COVID-19 mood disorders in this population may seem expected.

In the same way, melatonin levels -a product of serotonin- were also altered in patients with COVID-19 infection [33, 36] as well as migraine patients [37], explaining the high vulnerability for sleep disturbance when the two conditions coincide.

The post-COVID-19 headache includes a multifaceted spectrum of presentations, best described by Membrilla et al. [22] and Caronna et al. [38], including worsening of a pre-existing migraine, persistent headache after COVID-19 infection, and late-onset new persistent headache without a prior history of a primary headache disorder. Yet, these distinct patterns have not been obeyed by studies estimating the prevalence of headaches after COVID-19. Yet, the rate conveyed by our study (12% of the whole study population) fell within the range reported by these studies (10.6- 19.0%) assessed at the same post-COVID-19 period [11, 39, 40].

Hyperexcitability of the trigeminovascular system mediated by a sustained pro-inflammatory response during the acute infection may lead to central sensitization of second-order neurons, promoting the development of persistent headache after COVID-19 infection [22]. Further scientific efforts also consider an immune-mediated mechanism in the pathophysiology of post-COVID headaches based on a structural mimicry of the spike protein of SARS-CoV-2 with the CGRP receptor [41].

These justifications, combined with the pandemic's negative impact and fear of an unusually serious and potentially fatal illness, may also explain the higher frequency of depression, anxiety, insomnia, and headache exacerbation as post-COVID-19 symptoms in migraine patients [42, 43].

Another point worth discussing is that the prevalence of some neuropsychiatric symptoms in our study exceeded the range previously reported by epidemiological studies conducted before the pandemic among patients with episodic migraine. Chronic fatigue was previously estimated as (5.2%) [44], depression (5.2–17.2%) [44–46], and anxiety (13.4–18.8%) [44–46]. Such a notable increase in post-COVID-19 fatigue, depression, and anxiety warrants strenuous efforts to track these symptoms after recovery and to employ prudently directed rehabilitation programmes. On the other hand, the prevalence of other post-COVID-19 symptoms reported in our study fell within the previously reported range, such as insomnia (16–25.9%) [47, 48], pains (15.1–22.2%) [45, 49], and vestibular symptoms (12.2–17.8%) [49, 50].

In light of the previous findings, long-term rehabilitation policies should be employed to deliver appropriate care to migraine patients upon recovery from COVID-19 infection. Furthermore, the importance of the COVID-19 vaccine should be emphasized for individuals with migraine who appear to be vulnerable to the long-term consequences of COVID-19 infection.

The present study had some limitations that limited the generalizability of its results. Because this study targeted people who contracted COVID-19 during a single wave, the third wave, these findings are mainly limited to a specific viral variant that caused this wave. Also, most participants had a mild COVID-19 infection (*n* = 342, 83%8). Since the severity of COVID-19 infection strongly predicts post-COVID-19 symptoms, the current results can not be generalized. Furthermore, post-COVID-19 migraine chronification was based primarily on patients' self-report. Headache diaries might provide a more objective evaluation when headache characteristics of a pre-existing migraine were compared to those of the new headache pattern. Another limitation in this study was the marginal match between migraine patients and controls in age. Finally, adding another group of migraine patients not infected with COVID-19 to be compared to those who had contracted the virus might completely nullify the negative impact of the COVID-19 pandemic on migraine.

## Conclusion

Migraine patients are more likely to develop some post-COVID-19 symptoms than controls: anxiety (20.6 vs 7.4%), headache (17.6 vs 6.4%), insomnia (23.5 vs 12.3%), depression (20.6 vs 10.3%), fatigue (31.4 vs 21.6%), anosmia/hyposmia (19.1 vs 10.3%), and cacosmia (9.8 vs 3.9%). Rehabilitation strategies should be implemented to convey quality care to migraine patients upon recovery from COVID-19 infection.

## Data Availability

Authors report that the datasets used and/or analyzed during the current study are available from the corresponding author on reasonable request.

## Abbreviations

CDC: Centers for Disease Control and Prevention
COVID: Coronavirus disease
DN4: Douleur Neuropathique en 4 Questions
DSM-5: Diagnostic and Statistical Manual of Mental Disorders
FSS: Functional Status scale
ICHD-3: International Classification of Headache Disorders
IQR: Interquartile range
MoCA: Montreal Cognitive Assessment
NDPH: New daily persistent headache
PCCD: Post-COVID-19 cognitive dysfunction
RT-PCR: Reverse transcription-polymerase chain reaction
SARS-CoV-2: Severe acute respiratory syndrome coronavirus-2
VAS: Visual analogue scale
WHO: World Health Organization
